# TREATMENT OF PEDIATRIC FEMUR FRACTURES WITH FLEXIBLE STAINLESS STEEL INTRAMEDULLARY NAIL

**DOI:** 10.1590/1413-785220243203e267630

**Published:** 2024-08-02

**Authors:** Marcella Adryanne Dias Brandão, José Eduardo Sanches Arantes, Alceu José Fornari Gomes Chueire, Guaracy Carvalho

**Affiliations:** 1 Faculdade de Medicina de Rio Preto FAMERP, Faculdade de Medicina, Hospital de Base, Departamento e Ortopedia e Traumatologia, São José do Rio Preto, SP, Brazil.

**Keywords:** Stainless Steel, Child, Femur Fractures, Intramedullary Fixation of Fractures, Aço Inoxidável, Criança, Fraturas do Fêmur, Fixação Intramedular de Fraturas

## Abstract

Objectives: To identify the characteristics of patients and femur fractures treated with a stainless steel intramedullary nail (ESIN) in children under 15 years of age. Know the results of using the ESIN of related steel in the service. Methods: Retrospective study with review of hospital records and organization of data in spreadsheets. Result: 24 cases were identified, 17 male cases and 7 female cases. A minimum age of 4 years and a maximum of 11 years were observed (average of 7 years). The 3 most common trauma mechanisms were being run over (n:8, 33%) and falling from a height (n:8, 3%). The most common location of the fractures was in the mid-diaphyseal region (n: 20, 88%), only one case presented a bilateral femur fracture. The most common associated trauma was traumatic brain injury. The observation period observed several months between 2 and 5. With regard to complications, 3 cases were observed (12.5%) being bursitis, vicious construction and loss of reduction. Conclusion: Steel HIF shows similar good results. As the study includes the retrospective profile, the absence of a group and the small sample size. ***Level of Evidence IV, Case series.***

## RESUMO

Objetivos: Identificar as características dos pacientes e das fraturas de fêmur tratadas com haste intramedular flexível (HIF) de aço inoxidável em menores de 15 anos. A partir disso, conhecer os resultados relacionados ao uso da HIF de aço inoxidável no serviço. Métodos: Estudo retrospectivo, com revisão de prontuários hospitalares e organização dos dados em planilhas. Resultado: Identificados 24 casos, sendo 17 do gênero masculino e sete do gênero feminino. Foram observadas idade mínima de 4 anos e máxima de 11 anos (média de 7 anos). Os mecanismos de trauma mais comuns foram atropelamento (n: 8, 33%) e queda de altura (n: 8, 33%). A localização mais comum das fraturas foi na região médio diafisária (n: 20, 88%), apenas um caso apresentou fratura de fêmur bilateral. O trauma associado mais comum foi traumatismo crânio-encefálico. O período de consolidação observado variou entre 2 e 5 meses. No que se refere a complicações, foram observados três casos (12,5%), sendo estes: bursite, consolidação viciosa e perda de redução. Conclusão: A HIF de aço apresenta bons resultados, semelhantes aos das hastes de titânio. As limitações do estudo incluem o perfil retrospectivo, a ausência de grupo controle e o número pequeno da amostra. **Nível de evidência IV, Série de casos.**

## INTRODUCTION

 Trauma is a leading cause of morbidity and mortality in children and femoral fractures have a significant impact on the patients’ entire medical-familial care network. ^1^ Femoral diaphyseal fractures represent 1.4% to 1.7% of all fractures in the pediatric population, with males being the most affected. The incidence distribution is bimodal: first peak at 2-4 years of age and the second in adolescence. ^2^ Traffic accidents comprise the most frequent trauma mechanism, with the exception of children under 3 years of age. ^3^

 The preferred method for treating femoral fracture in children can vary according to age. ^4^ Conservative treatment with plaster after a period of traction has been one of the most used options in the past, but during the last decades new techniques have been developed. ^5^ For children aged 18 months to 6 years, conservative methods of treatment still predominate, represented by temporary traction and immobilization by pelvipodal plaster. ^6^ For children aged 6 to 16 years, fracture treatment depends on good reduction, not compromising areas of growth or blood irrigation and reducing complications, and it is possible to opt for elastic stable intramedullary nailing (ESIN) with favorable outcomes. ^1^ , ^6^ In addition, even for children aged under 6 years, there are current studies that recommend the use of ESIN and show good results. ^7^ Other surgical treatment methods include submuscular plates, external fixators and locked intramedullary nails. 

 The use of ESIN has reported complication rates ranging from 33-62%, mostly related to soft tissues with no need for surgical intervention, mainly involving pain and/or inflammation at nail insertion. ^8^

The objectives of this study were: trace the evolution of cases and complications of femur fractures treated with stainless steel elastic stable intramedullary nailing (ESIN) in children aged under 15 years. Find the outcomes related to the use of stainless steel ESIN in relation to titanium nailing.

## MATERIALS AND METHODS

This is a retrospective study with review of hospital records carried out in a reference Children and Maternity hospital in the non-metropolitan area of São Paulo. We analyzed data for the period from May/2016 to October/2020.

The present study was submitted to the Ethics Committee under opinion number 51363421.0.0000.5415 and its implementation was approved.

Inclusion criteria were: patients aged under 15 years, with a diagnosis of femur fracture, undergoing surgical treatment with stainless steel elastic stable intramedullary nailing (ESIN) technique. Exclusion criteria were: patients with follow-up for less than 12 months and pathological fractures. Patients with incomplete medical records were not included. The informed consent form was not required due to the study being only a review of medical records.

 The authors analyzed data from hospital medical records and used the following epidemiological information: gender, age, and trauma mechanism. Based on the initial radiographs, the fractures were classified according to fracture line location (diaphyseal or metaphyseal region), laterality and pediatric AO classification. ^9^

**Figure 1.**
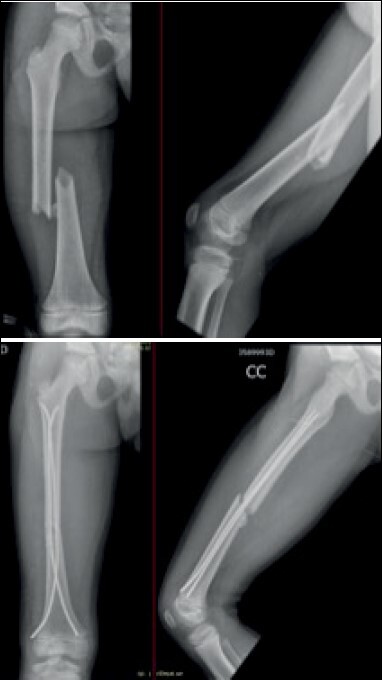
Immediate pre- and postoperative radiography of diaphyseal femur fracture.

 The surgeries were performed with the patient in horizontal dorsal decubitus on a radio-transparent operating table under general anesthesia. Fractures were reduced in a closed manner. Nail diameter was chosen to occupy 80% of medullary cavity diameter. The two nail were always chosen with the same thickness to avoid incorrect positioning and varus or valgus imbalance. The retrograde route was performed with two surgical steel flexible nails inserted with insertion point 2 cm proximal to the distal physis of the femur. The medial nail inserted up to 2 cm of the proximal femur and the lateral nail up to 1 cm of the greater trochanter. Skin incisions were made 3-4 cm distal to the planned insertion site depending on child size. The nails underwent molding with three times the diameter of the medullary channel, the arch vertex was placed at the level of the fracture zone. The nail was inserted with the aid of a “T” guide with the tip aligned with the medullary axis. The second nail was inserted by the same technique. The fracture was reduced and the nails were progressed. When the fracture was firmly fixed the rotation was checked before final anchoring. The nails had their end cut off and impacted. In cases with anterograde nails, the insertion was anterolateral in the subtrochanteric area. The nails were separated vertically by approximately 1–2 cm and horizontally by 0.5–1 cm. The reduction and positioning of the nails was checked by intraoperative scoping. Subcutaneous and skin were sutured with simple stitches ( Figure 1 ). 

**Figure 2.**
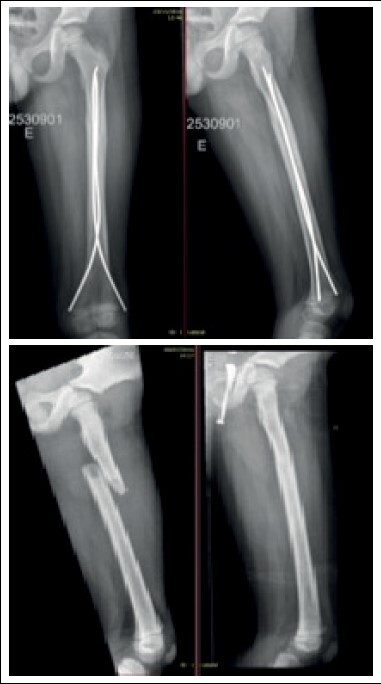
Preoperative and postoperative radiographs (4 months after fracture).

 In the immediate postoperative period, anteroposterior and lateral thigh radiographs were taken. Patients were discharged from hospital on the first day after surgery, except in some cases where polytrauma patients required further evaluation by other specialties. At discharge, patients were instructed to maintain zero load, keep the limb elevated and perform exercises to gain active and passive range of motion in the hip, knee and ankle joints. The first follow-up visit was one week later for surgical wound evaluation. The second follow-up visit was three weeks later for removal of stitches, control radiography. In the sixth week, new radiographs were taken and progressive load was allowed with the help and under the supervision of a physiotherapy team. Subsequently, patients had follow-up visits every two months for clinical and radiographic evaluation ( Figure 2 ). 

 Complications found included bursitis, vicious consolidation, and asymmetry of lower limbs (Clavien-Dindo type I and II). ^10 ^,^ 11^

**Figure 3.**
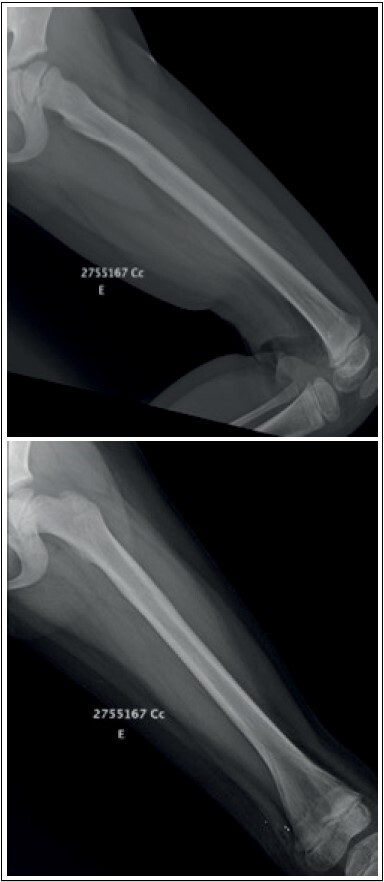
Radiography after nailing removal (12 months after surgery). Same patient as Figure 2 .

 Nailing removal was indicated to patients and their guardians after fracture consolidation, medullary canal reossification with return of the cortical layers of the femur, return of the ability to walk without pain, gain of range of motion and symmetrical muscle trophism to the contralateral limb. In selected cases, the removal procedure was performed in an operating room under general anesthesia with a one-day hospitalization ( Figure 3 ). 

## RESULTS

We traced 24 cases of femoral fracture treated with ESIN, of which 17 cases of males (71%) and 7 cases of females (29%), compatible with the distribution described in the literature. We observed a minimum age of 4 years and a maximum age of 11 years, with a mean of 7 years.

 The most common trauma mechanisms traced were being hit by a car (n: 8, 33%) and falling from a height (n: 8, 33%), followed by car accidents (n: 6, 25%). Two of the cases evaluated were related to objects falling on the lower limbs. The most common associated injury was traumatic brain injury traced in 4 cases, one of which required a neurosurgical approach ( Table 1 ). 

**Table 1.** Distribution of cases

Gender
Male: 17
Female: 7
Age
0 to 6 years: 9
7 to 10 years: 13
>10 years: 2
Trauma mechanism
Hit by a car: 8
Fall from a height: 8
Car accident: 6
Other (2)
Fracture level
Proximal metaphysis: 4
Diaphyseal: 20
Distal metaphysis: 0
Complications
Bursitis: 1
Asymmetry: 1
Vicious consolidation: 1

The most common fracture location was the mid-diaphyseal region (n: 20, 88%). Fractures were classified by AO criteria with 11 32-D/4.1 pattern fractures (42%) and 10 32-D/5.1 fractures. Four cases of fracture in the proximal metaphyseal region were observed. The left side was predominant, occurring in 13 cases (54.5%), only one case presented bilateral femur fracture.

The consolidation period ranged from 2 to 5 months, with an average of 2.9 months.

Regarding complications, 3 cases (12.5%) were observed. One case presented bursitis, another case had vicious consolidation and a third case had reduction loss.

ESIN removal occurred in 7 cases. However, 10 cases of the total had a loss of follow-up after 18 months, and it was not possible to determine the need for removal. These cases are represented by patients who did not return to the outpatient clinic for the scheduled visits.

## DISCUSSION

The definition of surgical treatment of femur fractures in children depends on variables such as age, patient weight, bone exposure or comminution, and availability of materials. For children aged 6–16 years, ESIN is an excellent treatment choice as it enables early load, quick return to activities of daily living, small incisions and low rates of serious complications.

 ESIN allows a slight movement in the focus of the fracture end, being beneficial for the formation of bone callus and characterizing the mechanism of relative stability, with the technique being widely used in the treatment of fractures of long limb bones, such as femur, tibia and humerus. ^12^

The treatment of femoral fractures in children is still the subject of discussions. The analysis of postoperative benefits makes ESIN a favorable option. Increasingly, our service opts for the use of ESIN as a therapeutic plan in cases with indication for such.

 The most common fracture patterns found in our study were simple transverse diaphyseal (46%) and simple oblique diaphyseal (42%). Buechsenscheutz et al. (2002) described 17 transverse fractures (41%) and 11 oblique fractures (26%), with 25 of the cases (60%) with diaphyseal line. ^13^ Hoffman in 2012 presented an index of 89.6% of single line fractures. ^2^

 In 1982 the concept of ESIN was introduced by a group in Nancy and described by Ligier in 1988. In this study, 123 fractures were observed in 118 children and 13 cases of superficial inflammation (9.8%) and 14 cases with deviation were identified (only one of the cases with rotational deviation). ^14^ Luhmann et al. ^15^ concluded that the most common complication was skin injury caused by the nails and it can be avoided by leaving only 2.5 cm of the nail external to the bone. They also observed that fixation of femoral fractures in overweight patients with ESIN is associated with increased sagittal angulation. ^15^ Flynn et al. ^16^ described in 2001 an analysis of 58 cases finding 7% of superficial infections and no cases of osteomyelitis. ^16^ Narayanan et al. ^17^ evaluated 78 cases of pediatric femoral fracture finding 58% of complications, the most common being pain or irritation at the nail insertion site. Poor consolidation was found in 8 cases, reduction loss in 5 cases, and refracture in 2 cases. Among these, 10 cases required re-approach. ^17^ In our study, the complication rate was 12.5%, one case of bursitis that occurred late due to friction of the prominent point of the lateral nail with the soft parts of the knee, requiring removal of nails that already had clinical radiographic criteria of consolidation. Another case with vicious consolidation evolves with valgus deviation of the femur and a third case with asymmetry of 4.0 cm, with conservative treatment by family decision. The case with reduction loss showed asymmetry of 4.1 cm between the lower limbs and epiphysiodesis in the contralateral distal femur was indicated. 

 The use of anterograde nail may present complications such as valgus deformity, narrowing of the femur and alteration in the epiphyseal plate. ^18^ In the study carried out, no lesion related to the use of anterograde nail was found in the analysis period. 

 Compared to other types of surgical approach such as submuscular plates, chosen in the treatment of fractures that present comminution of the femur diaphysis, heavier patients and fractures of the bone ends, ESIN presents good results with reduced operative time, less blood loss during surgery and lower cost of hospitalization. ^19^ The main limitations and difficulties in the use of flexible nails are found in patients with unstable, comminuted lines, in metaphyseal regions and in obese patients; however, there are new articles that even in these cases ESIN can be well indicated. ^8^ In relation to external fixation of closed fractures, ESIN is associated with earlier load, better range of motion, faster return to activities of daily living and lower incidence of discrepancy in limb length. ^20^ Our patients presented no case of refracture, even in cases where the nails were removed, demonstrating a positive result in relation to a previous study carried out in our service with a rate of 17% of refracture with the use of an external fixator. ^21^

 Differently from what is found in much of the literature on ESIN, the material of the nails available in the service is stainless steel. Wall et al. ^22^ showed that the rate of major complications (for example, irritation caused by nail requiring revision surgery, infection, delayed union, nail breakage) was higher in titanium nails than in stainless steel nails (35.7% and 16.7%, respectively). ^22^ In a study also in 2008 Soni et al. ^23^ evaluated two-dimensional computerized models with qualitative analysis favorable to the use of titanium nails, but quantitative analysis with statistically similar values between titanium and steel. ^23^ Comparative studies with results of equivalent or even superior quality for the use of steel nails were found. In addition, the cost of steel ESIN can be up to three times lower than titanium ESIN. ^24^ , ^25^

 There is no agreement in the literature regarding the removal of implant material, and it is mentioned that it is necessary only in symptomatic patients or when the implant may compromise the physis. ^26^ In our service, the nails were removed in 7 patients, but in 10 cases of the total there was loss of follow-up after 18 months and it was not possible to trace the need for nail removal. 

The study presents the bias of loss of follow-up, as some patients do not return for scheduled visits. In addition, there was a large number of incomplete medical records, which leads to a reduced total number for analysis.

## CONCLUSION

Our service uses steel ESIN and presents good results compatible with the literature. Study Limitations include the retrospective profile with insufficient information in some medical records with loss of follow-up, the lack of a control group, and the small sample size. In addition, formal measurements of angular changes were not performed in all patients, only in patients with clinical complaints. The complication rate found was lower than that presented in the literature. We conclude and demonstrate with our described cases that the use of stainless-steel elastic stable intramedullary nailing is an option that is viable, cheaper, with a low complication rate, and accessible for the treatment of pediatric patients with diaphyseal femur fracture.
